# The Promise of Automated Home-Cage Monitoring in Improving Translational Utility of Psychiatric Research in Rodents

**DOI:** 10.3389/fnins.2020.618593

**Published:** 2020-12-17

**Authors:** Alfred Mingrone, Ayal Kaffman, Arie Kaffman

**Affiliations:** ^1^Department of Psychology, Southern Connecticut State University, New Haven, CT, United States; ^2^Department of Psychiatry, Yale University School of Medicine, New Haven, CT, United States

**Keywords:** automated home cage monitoring, rodents, translational research, psychiatry, Intellicage system, PhenoTyper, Actual-HCA, Chora feeder

## Abstract

Large number of promising preclinical psychiatric studies in rodents later fail in clinical trials, raising concerns about the efficacy of this approach to generate novel pharmacological interventions. In this mini-review we argue that over-reliance on behavioral tests that are brief and highly sensitive to external factors play a critical role in this failure and propose that automated home-cage monitoring offers several advantages that will increase the translational utility of preclinical psychiatric research in rodents. We describe three of the most commonly used approaches for automated home cage monitoring in rodents [e.g., operant wall systems (OWS), computerized visual systems (CVS), and automatic motion sensors (AMS)] and review several commercially available systems that integrate the different approaches. Specific examples that demonstrate the advantages of automated home-cage monitoring over traditional tests of anxiety, depression, cognition, and addiction-like behaviors are highlighted. We conclude with recommendations on how to further expand this promising line of preclinical research.

## Introduction

Rodents are the most commonly used animal models for studying the biological underpinning of psychiatric conditions and the development of novel pharmacological treatments (Richardson, 2015; Ellenbroek and Youn, 2016). However, most drugs that show promise in preclinical studies fail in clinical trials, raising serious concerns about the utility of work in rodents to identify novel pharmacological interventions (Miller, 2010; O’Brien et al., 2014; Kaffman et al., 2019). One possible reason for this failure is the overreliance on behavioral tests that are brief (e.g., 5–10 min) and highly sensitive to external conditions including lighting, noise, smell, or time of day. Furthermore, for convenience, most behavioral tests are conducted during the light phase when rodents are inactive. Human-related factors such as sex of the tester, experience handling rodents, and previous contacts with the animals can also impact behavioral outcomes in these tests (Jhuang et al., 2010; Richardson, 2015; Bains et al., 2018; Balzani et al., 2018). The short duration of current tests hinders their use for longitudinally assessing responses to chronic drug administration in a manner that mimics clinical trials (Richardson, 2015; Bains et al., 2018; Kaffman et al., 2019). Thus, the brevity of the sampling and their sensitivity to external variables make the outcomes of current tests difficult to replicate and unsuitable for long prospective pharmacological studies.

The central proposition of this review is that expanding the use of automated home-cage monitoring (AHCM) will improve construct and clinical validity of psychiatrically relevant research in rodents. Although some excellent reviews have described the utility of AHCM (Jhuang et al., 2010; Richardson, 2015; Bains et al., 2018; Balzani et al., 2018; Kiryk et al., 2020), they did not focus on translational psychiatric research. Moreover, rapid expansion of commercially available systems, and the number of transgenic rodents with clinically relevant mutations, merits an updated review.

Automated home-cage monitoring records rodent behavior in their home cage over extended periods using minimal human contact. The large dataset generated by AHCM creates a robust behavioral profile for individual or grouped-house animals that spans circadian and estrous cycles and cover extended periods. This experimental design is particularly helpful for monitoring response to pharmacological interventions that better mimics human clinical trials (Robinson and Riedel, 2014; Goodwill et al., 2018; Kaffman et al., 2019; Kiryk et al., 2020). AHCM provides information about behavioral changes when animals are more active or exposed to stress, increasing their sensitivity to identify subtle changes (Bains et al., 2018). The use of radio frequency identification system (RFID) enables behavioral tracking of individual animals that are housed together, providing important information about the impact of social context on complex behavior and learning (Bains et al., 2018; Balzani et al., 2018; Kiryk et al., 2020). The large number of observations obtained using AHCM reduce behavioral noise associated with brief testing and improve reproducibility of behavioral data within and across labs (Lipp et al., 2005; Krackow et al., 2010; Robinson et al., 2018; Prevot et al., 2019).

In this review we describe the three most common AHCM approaches in rodents: operant wall systems (OWS), computerized visual systems (CVS), and automatic motion sensors (AMS). We review the pros and cons for each system, describe how these approaches are integrated, and provide examples of relevant success stories. We conclude with a short summary and suggestions for future directions.

## Operant Wall Systems

Operant wall systems are equipped with 2–3 small openings (aka hoppers) that detect nose pokes using an infrared beam and deliver food pellets or liquid in response to learned behaviors. Behaviors are directed by light or tone cues controlled by the operant wall. For example, rodents in the “*switch task*” learn to associate a short duration of light (3 s) with a nose poke in the left hopper and a long duration of light (6 s) with poking the right hopper. Only correct first responses to the appropriate stimuli deliver a reward. Poking the right hopper in response to short light stimuli is considered an error used to construct a learning curve. A key measurement of this task is the time it takes the animal to start poking the right hopper in response to longer duration of light. This “switch” requires time-keeping and the ability to distinguish between short and long-cues (Balzani et al., 2018).

A common commercial OWS is the Chora Feeder (AM Microsystems). Up to 250 units can be connected and processed by a single computer, allowing for rapid data collection from many animals. OWS assess several psychiatrically relevant outcomes in rodents including working memory, flexibility, time-keeping, and feeding behavior (Balzani et al., 2018). Task-specific learning speeds and error rates are updated every 10 ms across the entire circadian cycle (see Table 1 for summary).

**TABLE 1:** Pros and cons of the Chora wall feeder, IntelliCage, PhenoTyper, and Actual-HCA systems.

System/company/institution	Key features	Pros	Cons	References
Chora feeder – AM Microsystems and the Instituto Italiano di Tecnologia	• Simple automated wall feeder	• More affordable (about $5,000 per unit) • Up to 250 Chora feeders can be connected and processed simultaneously to a single computer • Can assess working memory, feeding, flexibility, time-keeping over an extended period of time • Compatible with other Chora-brand modules • Measurements are refreshed every 10 ms providing high temporal resolution of behaviors	• Likelihood of device malfunction increases over time, may result in data loss or removal of animals from study • Dispensers need to be cleaned, requiring down-time (data loss) and unintentionally subjecting rodents to extinction • Requires single housing	• Tucci et al., 2014 • Balzani et al., 2016 • Lassi et al., 2016 • Balzani et al., 2018
IntelliCage – TSE Systems	• RFID transponders and computerized regulated access to operant conditioning corners for a group of up to 16 rodents	• Social grouping for extended period of time • Individual mice can be identified using RFID • High flexibility in controlling access to liquid including timing, place, cue-mediated consumption, and punishment with an air puff. • Automated data acquisition • Large amount of behavioral data for up to 16 mice on anxiety, anhedonia, compulsive drug consumption, spatial, reversal and episodic memory • Capable of assessing social hierarchy, social learning, and social affinity • Good replicability across labs • Compatible with chronic drug administration • Highly modular and accessorized for additional features such as running wheel or social chamber	• No visual tracking info • High upfront cost ($65 k for one cage), but cost needs to be also considered in the context of the large number of rodents that can be tested at the same time. • Work with males is possible but more challenging due to aggression • No information on social interaction between specific individuals • Complicated statistical analysis and need to address/adjust for multiple testing	• Safi et al., 2006 • Branchi et al., 2010 • Radwanska and Kaczmarek, 2012 • Nowak et al., 2013 • Benner et al., 2014 • Masuda et al., 2016 • Alboni et al., 2017 • Dere et al., 2018 • Voikar et al., 2018 • See Kiryk et al., 2020 for an excellent review
PhenoTyper – Noldus Information Technology	• Top-down infra-red video recording linked to trainable visual system	• Highly accurate information about locomotor activity • Relatively low cost when purchasing multiple boxes (38,000 for four boxes) • Has been successfully used to assess anxiety (spot-light test) • Can be easily accessorized to add running wheel, lickometer, and operant learning wall • Compatible with optogenetics and calcium imaging • Reliable data across labs	• Single housing and thus relatively low behavioral throughput • Relatively bulky cage that requires large room to house multiple units • Camera provides top-down view that limits finer behavioral assessment	• Aarts et al., 2015 • Robinson et al., 2018 • Logan et al., 2019 • Prevot et al., 2019
Actual-HCA (Home Cage Analyzer) – Actual Analytics	• Visual monitoring with an infra-red camera that sits alongside the home cage and a RFID transponder system for monitoring position of multiple rodents in a standard housing cage	• Detailed visual information integrated with positional information for group housed animals • Can be used to assess social proximity • RFID system can also provide information on body core temperature • Compatible with standard IVC racks	• Not cheap (about $17,000 per cage) • Requires two IVC rack spaces per cage	• Bains et al., 2016 • Tse et al., 2018 Mitchell et al., 2020a • Mitchell et al., 2020b

Several examples highlight the utility of OWS in psychiatric research. Tucci et al. (2014) have shown that mice with a mutated β-catenin fail to show the normal cognitive improvement seen during the dark-phase of the circadian cycle in wild-type littermates. This finding demonstrates the utility of OWS to map circadian-specific cognitive deficits that would probably be missed if the animals would be tested during the light cycle as routinely done during conventional testing. Further, cognitive deficits seen in individuals with *de novo* genetic mutations in the β-catenin gene (Tucci et al., 2014) may also be more pronounced during a specific time in the day, allowing for the development of more effective interventions. A similar approach has shown that mice with a mutation in the clock gene Zfhx3 have abnormally short circadian periods and are impaired in their ability to track time in short-term cognitive tasks (Balzani et al., 2016). Hyperphagia is commonly seen in individuals with Prader-Willi Syndrome and Lassi et al. (2016) used OWS to identify short and long-term abnormalities in food anticipatory behavior in Prader-Willi mice (Lassi et al., 2016). These examples, require repeated testing over an extended period of times that could not be attained using brief conventional behavioral testing.

OWS has several important limitations. For example, the likelihood of device malfunction increases over long durations leading to data loss and potentially removing animals from the study. Dispensers also require cleaning which can result in data loss during down-time, as well as unintentionally subjecting rodents to extinction training. Finally, most systems require that animals be housed individually which is stressful and unnatural for rodents, an issue that has been addressed by coupling RFID with specialized corner feeders using the IntelliCage (see below).

### The IntelliCage System

**FIGURE 1:**
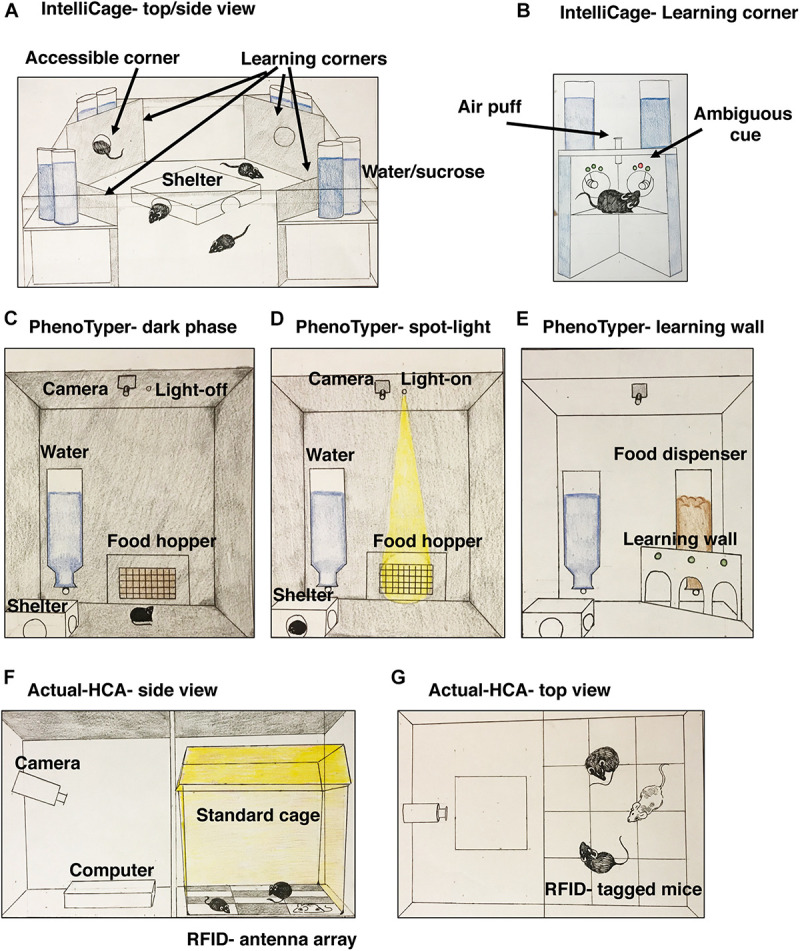
A Schematic illustration of the IntelliCage system **(A)** with a close-up view of one operant conditioning corner **(B)**. The IntelliCage system uses RFID transponders and computerized regulated access to operant conditioning corners for a group of up to 16 mice where individual behavior is assessed. A simplified version of the PhenoTyper and its use for the spot-light test of anxiety **(C,D)** and a possible addition of an operant learning wall **(E)**. Unlike the IntelliCage and the Actual-HCA systems, behavior in the PhenoTyper is usually conducted in individually housed rodents. The Actual-HCA system uses a combination of visual monitoring with an infra-red camera and a RFID transponder system to monitor the positions of multiple mice in a standard housing cage **(F,G)**.

The IntelliCage (TSE Systems) is a large cage (62 cm × 44 cm × 21 cm) with four computer-controlled operant conditioning corners (OCC). Up to 16 mice can be housed in one IntelliCage, each carrying a RFID transponder that identifies animals via antennae located in each OCC. Only one mouse can enter the OCC, and a computerized door grants access to specific mice during specific times (Figure 1). When a mouse enters, it faces two operant conditioning walls arranged at 90 degree-angles. Each operant conditioning wall has three LED-lights above an infra-red monitored nosepoke hole that can be blocked or opened by the computer to provide access to a water bottle equipped with lickometer. A device that delivers air-puffs is placed at the top of the OCC and can “punish drinking” and establish conflict-avoidance (Figure 1). The spatial arrangement of the OCC and the ability to control their access allows one to study spatial learning (e.g., water is available only at the East corner of the cage), reversal learning (e.g., water is now available at the West corner) and episodic memory (e.g., water is available at the East corner but only between 18:00–20:00). Having two bottles also allows one to assess hedonic behavior (preference for sweetened water), neophobia (i.e., latency entering a corner), and defensive or compulsive behaviors after being startled by an air-puff. Different colored LEDs can assess response to ambiguous-cue, where green color signals safety, red signals punishment with an air-puff, and the co-presentation of both serves as an ambiguous cue (Figure 1). The Intellicage allows direct comparisons between individuals from different conditions that are housed together over an extended period of time. This feature is not available in conventional testing, providing new opportunities to study social hierarchy and response to social instability in rodents (Branchi et al., 2010; Nowak et al., 2013; Benner et al., 2014).

Several studies demonstrate the potential of the IntelliCage system to transform preclinical psychiatric research. For example, treatment of mice exposed to chronic stress led to faster improvement in anhedonic measure at one-week, but not at 3 weeks post stress when compared to vehicle treated mice. Interestingly, response to ambiguous cue was more “optimistic” in stressed mice treated with fluoxetine compared to stressed mice treated with vehicle alone (Alboni et al., 2017). These findings suggest that SSRI transiently accelerate hedonic recovery from stress, but more importantly lead to stable cognitive/behavioral response to ambiguous cue (Alboni et al., 2017). This is consistent with growing clinical data suggesting that SSRI induce a more “rosy perception” (Michely et al., 2020) and provide a novel experimental paradigm to study this phenomenon in mice.

Safi et al. (2006) showed that diazepam injection reduces anxiety-like behavior. In this paradigm, conflict avoidance is established by punishing a water-deprived mouse with an air-puff when first approaching the drinking corner (Safi et al., 2006). This approach differs from conventional tests of anxiety in that it examines behavior over an extended period of time. Moreover, the avoidance conflict can be repeatedly tested across multiple days. This allows for a cross-over design in which avoidance behavior is tested after administration of diazepam, saline, or no injection in the same animal. This is difficult to do using conventional tests of anxiety such as the open field test and elevated plus maze that rely heavily on initial avoidance of novelty that is significantly diminished with re-exposure (Safi et al., 2006).

The IntelliCage was also used to assess compulsive alcohol consumption (Radwanska and Kaczmarek, 2012), characterize cognitive and social deficits in mouse models of Alzheimer’s disease and autism spectrum disorder (Masuda et al., 2016; Dere et al., 2018), identify important sex differences in complex behavior (Dere et al., 2018), and characterize behavioral deficits in animals with lesions to the hippocampus and prefrontal cortex (Voikar et al., 2018). The main drawbacks of the IntelliCage include its upfront cost, the absence of visual information about in-cage behavior, and the lack of information about social interaction between individuals (see also Table 1).

## Computerized Visual Systems

Infrared cameras with trainable CVS have been used by several groups (Jhuang et al., 2010; Goodwill et al., 2019; Singh et al., 2019). This approach has benefited from tremendous technological progress in trainable computational software for detecting motion (Jhuang et al., 2010) and the increased affordability of small, high-resolution infrared cameras. Indeed, Singh et al. (2019) reported the use of affordable small infra-red cameras that are mounted on cages and automatically analyzed using open-source software (Singh et al., 2019). CVS monitor behavior at high temporal resolution over extended periods including the dark and light phases of the circadian cycle, and trainable software are publicly available (Bains et al., 2018). The trainable nature of CVS allows for extensive flexibility in terms of the behavioral phenotypes it monitors (Jhuang et al., 2010; Bains et al., 2018). Further, the accuracy of CVS is on par with human scoring while eliminating possible human bias (Roughan et al., 2009; Jhuang et al., 2010; Tse et al., 2018). Some examples of behavioral features that can be assessed include locomotor activity, avoidance behavior, grooming, stereotypy, climbing, and feeding (Roughan et al., 2009; Jhuang et al., 2010; Goodwill et al., 2019; Prevot et al., 2019). CVS can also reliably assess responses to postoperative pain and analgesic treatment (Roughan et al., 2009), sleep patterns (Brown et al., 2016), and distinguish REM from non-REM sleep (McShane et al., 2012).

Several commercial CVS including PhenoTyper (Noldus) and Actual-HCA (Home cage analyzer) show significant promise (see Figure 1 and Table 1). The PhenoTyper is a large box (45 × 45 × 60 cm) with an infrared camera on top linked to trainable visual software, a food hopper, shelter, and water access. Measurements of locomotor activity obtained using the PhenoTyper show good reproducibility across labs (Robinson et al., 2018) and a light at the top of the cage can be programmed to illuminate the food hopper during specific periods of the dark phase (Figure 1B). This simple manipulation known as the light-spot test has been successful for assessing anxiety-like behavior (Aarts et al., 2015; Prevot et al., 2019). The basic PhenoTyper can be modified to include an operant wall, lickometer, and running wheel and is compatible with optogenetics and calcium imaging.

Computerized visual systems helped identify depression-like markers in female mice exposed to early life stress (Goodwill et al., 2019). These included reduced locomotor activity and lower frequency of self-grooming recorded over a period of 5 days that were reversed after a single injection of Ketamine (Goodwill et al., 2019). The ability to measure depression-like behavior in the home cage over an extended period of time demonstrates the advantage of this approach compared to outcomes obtained by more conventional brief tests such as the forced swim test and the tail suspension test. Moreover, given the transient nature of Ketamine response in humans (Marcantoni et al., 2020), it would be of great interest to clarify whether the response to Ketamine persists 2 weeks after administration in mice exposed to early life stress.

Using the PhenoTyper light-spot test, Prevot and colleagues found robust avoidance behavior after light exposure in mice exposed to two different types of chronic stress (Prevot et al., 2019). Unlike the robust behavioral phenotype seen with the spot-light test, anxiety-like behavior assessed using a more traditional tests such as the open field, elevated plus maze, novelty suppressed feeding failed to show consistent increase in anxiety in these two stress paradigms (Prevot et al., 2019). This is likely because the light spot induces an avoidance conflict during the dark phase when the animals are more active and because this behavioral response is monitored over 4 h in the complete absence of human presence. These findings highlight the advantages of AHCM over traditional behavioral tests for measuring stress-induced anxiogenic behavior.

Limitations of the CVS include the requirement for adequate contrast between the rodent and its background and the need for most systems to house animals individually (see ActualHCA below for an important exception). Furthermore, using trainable software is not trivial and requires significant computational expertise and initial manual validation. Similarly, assembling homemade cameras such as the one described by Singh et al. (2019) requires some expertise, has relatively low resolution, and is vulnerable for data loss due to technical glitches.

## Automatic Motion Sensors

AMS use motion sensors to assess locomotor activity. Some systems install an electronic sensor board underneath the home-cage with planar-sensing electrodes that track movements across the array (Pernold et al., 2019). Other approaches use infrared beams to track activity or distance traveled on a running wheel. AMS generate data about distance traveled, velocity, and time spent at specific locations within the cage. They are unobtrusive and are not affected by lighting conditions, allowing for reliable data collection during the dark cycle on locomotor activity and sleep behavior (Brown et al., 2016). Most AMS systems provide relatively limited information on locomotor activity for group-housed animals. This, however, is not the case for the Actual-HCA system (Actual analytics). In the Actual-HCA system, mice are individually tagged with RFID transponders that communicate with a sensor board placed underneath a standard cage tracking the exact location of each rodent. A high-resolution infrared camera is linked to the sensor board providing visual information that is analyzed using sophisticated trainable software (Figure 1C). This unique combination allows researchers to automatically assess proximity between cage-mates and to visually monitor social behavior during specific times. Although convincing data about the utility of the Actual-HCA to detect social deficits in rodents is still tenuous (Mitchell et al., 2020b), it was successfully used to characterize social behavior in rats administered Phencyclidine (Mitchell et al., 2020a). The Actual-HCA provided important information about sedative and latent effects of treatment with chlorpromazine, clonidine and amphetamine that were not detected using traditional manual scoring (Tse et al., 2018). The use of a running wheel is a particularly interesting example of AMS because it allows one to examine voluntary behavior with hedonic value that has been used to map molecular details regarding the circuitry that program runner’s high (Fernandes et al., 2015). Running wheels have also been used to assess procedural learning (i.e., running with missing rungs) and the role that myelin plays in this type of plasticity (McKenzie et al., 2014; Xiao et al., 2016). Disadvantages of AMS include high cost for commercial systems and the relatively limited psychiatrically relevant information available from systems that rely exclusively on motion sensors (see Table 1).

## Conclusion and Future Directions

The ability of AHCM to collect complex behavioral data at high temporal resolution over extended periods with minimal human interference makes it a promising frontier for improving translational psychiatric research in rodents. AHCM provides robust behavioral data that is less sensitive to erratic transient environmental cues and can identify behavioral changes specific to dark or light phases of the circadian and menstrual cycles. Longer observation also provides a more appropriate method for assessing rate of response and efficacy of pharmacological interventions. Several examples are now available to demonstrate the advantages of AHCM over traditional behavioral tests for anxiety (Safi et al., 2006; Prevot et al., 2019), response to antidepressants (Alboni et al., 2017; Goodwill et al., 2018), compulsive alcohol consumption (Radwanska and Kaczmarek, 2012), cognitive deficits in Alzheimer disease (Masuda et al., 2016) and overall reproducibility across labs (Lipp et al., 2005; Krackow et al., 2010; Robinson et al., 2018).

Deciding which system to use depends on the question at hand and practical considerations such as cost and specific expertise within each lab. For example, CVS offers high flexibility for monitoring specific micro-behaviors such as grooming, climbing, nesting, and stereotypic behaviors whereas OWS provides valuable information about impulsivity, attention, motivation, time keeping, and working memory that are highly relevant for psychiatric research. AMS is a relatively simple method to assess general activity in rodents, but overall provides less relevant information for psychiatric research. Several commercial systems now integrate the different approaches (i.e., OWS, CVS, and AMS) and provide tremendous flexibility in terms of testing individual rodents in group settings (Figure 1).

In terms of current challenges and future directions, additional effort is needed to develop user-friendly/affordable AHCM systems and to establish forums for sharing technologies and training. This effort should focus on testing reproducibility across labs and collaboration between academic research and pharmaceutical companies. This collaboration will be especially valuable for testing pharmacological interventions in mouse models of early life stress, compulsive drug-seeking behavior, and transgenic animals with mutations in genes implicated in schizophrenia, autism- spectrum disorder, and Alzheimer’s disease. Additional studies are needed to characterize behavioral and cognitive changes during the juvenile period, changes associated with aging, and sex differences. Whether AHCM will indeed improve the predictive validity of translational work in rodents is yet to be clarified. In this regard, re-evaluating outcomes of previous promising preclinical outcomes in rodents that later failed in clinical trials would perhaps be the most compelling way to demonstrate the advantage of the AHCM approach over more conventional tests. Some important examples include the use of corticotrophin releasing factor receptor one antagonists for anxiety (Kaffman et al., 2019), mGluR5 antagonists to rescue cognitive and social deficits in fragile X syndrome knockout mice (Bhakar et al., 2012; Berry-Kravis et al., 2016), and the use of N-acetylcysteine for drug addiction (Nocito Echevarria et al., 2017; Spencer and Kalivas, 2017). Finally, the next generation of AHCM should combine behavioral outcomes with electroencephalography, *in vivo* electrophysiology, calcium imaging, and optogenetics/chemogenetics manipulations to gain additional insights regarding the underlying circuits that drive behavioral outcome.

## Author Contributions

AM reviewed the literature, wrote, and edited the manuscript. AyK, made the illustrations and edited the manuscript. ArK, conceptualized the content, reviewed the literature, wrote, and edited the manuscript. All authors contributed to the article and approved the submitted version.

## Conflict of Interest

The authors declare that the research was conducted in the absence of any commercial or financial relationships that could be construed as a potential conflict of interest.
